# Control of cell cycle entry and exiting from the second mitotic wave in the *Drosophila *developing eye

**DOI:** 10.1186/1471-213X-8-7

**Published:** 2008-01-24

**Authors:** Madina J Sukhanova, Wei Du

**Affiliations:** 1 Ben May Department for Cancer Research, the University of Chicago, 929 East 57th Street, Chicago, IL 60637, USA

## Abstract

**Background:**

In the morphogenetic furrow (MF) of the *Drosophila *developing eye, all cells arrest in G1 and photoreceptor cell differentiation initiates. As the cells exit the MF, Notch signaling is required for the uncommitted cells to enter a synchronous round of cell division referred to as the "second mitotic wave" (SMW). How cell cycle entry and exit in SMW is regulated remains unclear. Recent studies have suggested that Notch signaling controls S phase in the SMW by regulating Cyclin A and the E2F transcription factor independent of Cyclin E. In this manuscript, we investigate the developmental regulation of cell cycle entry into and exit from SMW.

**Results:**

We demonstrate here that Cyclin E-dependent kinase activity is required for S phase entry in SMW. We show that removal of *Su(H)*, a key transcription factor downstream of Notch signaling, blocks G1/S transition in SMW with strong upregulation of the Cyclin E/Cdk2 inhibitor Dacapo (Dap). We further show that the upregulation of Dap, which is mediated by bHLH protein Daughterless (Da), is important for cell cycle arrest of *Su(H) *mutant cells in SMW. Finally we show that removal of Dap leads to additional cell proliferation and an accumulation of the non-photoreceptor cells in the *Drosophila *developing eye.

**Conclusion:**

Our data demonstrate that Cyclin E/Cdk2 kinase activity is absolutely required for S phase in SMW, and that Dap is required for the proper cell cycle arrest of cells exiting the SMW. In addition, our results suggest that the G1 arrest of *notch *and *Su(H) *mutant cells in the SMW are regulated by distinct mechanisms, and that the upregulation of Dap contributes the G1 arrest of *Su(H) *mutant cells.

## Background

Although cell cycle regulation is well characterized in single cell organisms or in tissue culture settings, much less is known about the control of cell proliferation during the development of multicellular organisms, in particular how developmental signals are connected to the cell cycle machinery to coordinate cell proliferation with differentiation. The *Drosophila *developing eye is an excellent model system that has been extensively used to dissect the developmental control of cell proliferation.

The *Drosophila *compound eye is composed of about 800 repeating units, or ommatidia. Each ommatidium contains eight photoreceptor cells (R1–8), surrounded by bristle, cone and pigment cells. Photoreceptor differentiation begins during the last larval instar within the morphogenetic furrow (MF). During eye development, the MF sweeps across the eye disc from posterior to anterior. All of the cells in the MF and immediately anterior to the MF arrest in G1 [1,2]. Cells that emerge from the posterior of the MF can be divided in two subpopulations: cells in preclusters, which will begin neuronal specification and exit cell cycle, and undifferentiated cells surrounding the preclusters, which will enter a synchronous round of cell cycle, the SMW [3]. The SMW is important to generate a pool of undifferentiated cells, which can be recruited into the differentiating ommatidia [4].

Notch signaling plays multiple roles in regulating cell proliferation and differentiation in the developing eye disc [5-8]. Initially, Notch signaling is required for the upregulation of the proneural gene Atonal (Ato) through removal of the inhibitory function of the downstream transcription factor Su(H) [5,8]. Subsequently, Notch signaling is required to limit the number of cells that will differentiate into photoreceptors through a Su(H)-dependent process called "lateral inhibition". Consistent with this, while *notch *mutant cells block photoreceptor cell differentiation, a majority of the cells within the *Su(H) *mutant clones near the MF differentiate as a photoreceptors [5]. Notch signaling was also shown to be required for S phase entry in the SMW [9,10]. Inhibition of Notch signaling, either by mutation of Notch receptor or by mutation of Su(H), blocked S phase in the SMW [9,10]; however, the mechanism was not clear. It is possible that distinct mechanisms are involved in the cell cycle arrest in the absence of the Notch receptor or the Su(H) transcription factor, given their different effect in photoreceptor differentiation. As high levels of Cyclin E protein were observed in the both the *notch *and the *Su(H) *mutant cells blocked in G1, it was suggested that Cyclin E function was not involved in Notch signaling mediated cell cycle regulation in SMW [9,10]. However, since Cyclin E functions through regulating the activity of its partner Cdk2 and since the Cyclin E/Cdk2 kinase activity can also be inhibited by p21/p27 family of cdk inhibitor Dacapo (Dap), the protein level of Cyclin E does not always correlate with the activity of Cyclin E/Cdk2 kinases. In fact, overexpression of Dap, which inhibits Cyclin E-dependent kinase activity, also induced Cyclin E expression and protein accumulation [11,12].

Dap is the only Cdk inhibitor identified in *Drosophila *and was shown to be specific for the Cyclin E-dependent but not Cyclin A, B or D-dependent kinases [11,13]. Dap expression parallels the exit of cells from the cell cycle in embryos and mutation of *dap *leads to an extra division during embryogenesis [11,14]. In the developing eye disc, Dap is expressed in the MF and the SMW, where cells have either exited or will be exiting the cell cycle. Dap-HB, an enhancer that drives Dap expression specifically in the photoreceptor R2 and R5 precursors, was shown to be directly regulated by EGFR signaling and bHLH proteins Ato and Da, which are the same developmental cues that control the differentiation of these two photoreceptors [15,16]. Surprisingly, removing Dap in the developing eye disc did not show dramatic alteration in the pattern of cell proliferation and did not have a dramatic effect on the adult eye phenotype [9,11,17]. These observations have led to the idea that Dap does not have a role in regulating the cell cycle in the developing eye.

In this manuscript, we investigate the role of Cyclin E/Cdk2 kinase activity in the SMW, the inability of *Su(H) *mutant cells to enter S phase in the SMW, and the role of Dap in cell cycle regulation in the developing eye. We show that *cdk2 *mutant cells accumulated high levels of Cyclin E but failed to enter S phase in the SMW, demonstrating an absolute requirement of Cyclin E/Cdk2 kinase activity for S phase entry in the SMW and a lack of correlation between the level of Cyclin E protein and Cyclin E/Cdk2 kinase function. In addition, we showed that the G1 cell cycle arrest of the *Su(H) *mutant cells is mediated in part by the upregulation of Dap and can be overcome by simultaneous expression of both Cyclin E and Cdk2. The upregulation of Dap in *Su(H) *mutant cells was dependent upon the basic helix-loop-helix (bHLH) proneural protein Da, suggesting a tight link between the role of Su(H) in cell type specification and in S phase regulation in SMW. The important role of Dap in mediating G1 arrest of *Su(H) *mutant cells prompted us to investigate the role of Dap in cell cycle regulation during normal eye development. Contrary to previous reports that suggested that there was no cell cycle consequence of loss of Dap in the developing eye [9], we show that Dap is required for the precise cell cycle exit from the SMW and that loss of Dap leads to the appearance of extra accessory cells in the developing pupae retina.

## Results and discussion

### cdk2 mutant cells accumulate high levels of CycE and are blocked from S phase entry in the SMW

**Figure 1:**
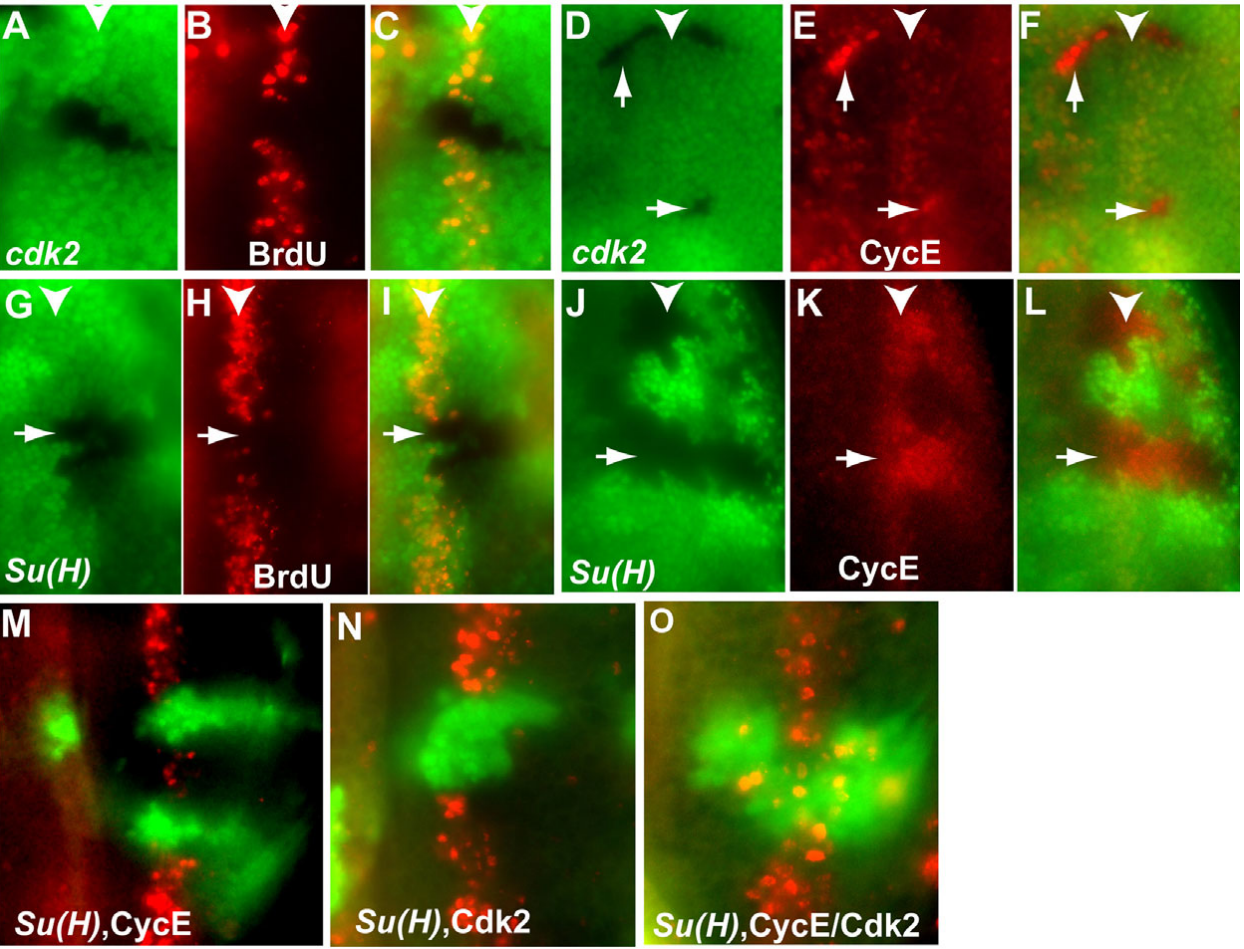
**Cyclin E/Cdk2 activity is required for S phase in SMW and is sufficient to induce S phase in *Su(H) *mutant cells**. (A-C) BrdU (red) incorporation is blocked in *Minute*^+^*cdk2 *mutant cells in the SMW. (D-F) In *Minute*^+^*cdk2 *mutant cells Cyclin E level (red) is up-regulated. (G-I) In *Su(H) *mutant cells, BrdU (red) is not incorporated in the SMW. (J-L) *Su(H) *mutant cells have high level of Cyclin E protein (red). Mutant clones were marked by the absence of GFP (A-L). In all discs, anterior is to the left. White arrowheads in these and subsequent figures indicate the position of the SMW. The over-expression of Cyclin E (M) or Cdk2 (N) alone is not able to induce ectopic BrdU incorporation (red) in *Su(H) *mutant clones. (O) Simultaneous over-expression of Cyclin E and Cdk2 induces large number of BrdU incorporation in *Su(H) *mutant cells. MARCM clones were marked by the GFP (M-O).

To determine if Cyclin E/Cdk2 activity is required for S phase entry in the SMW, we tested the cell cycle effect of removing Cdk2, the cdk partner of Cyclin E in *Drosophila *[13]. As expected *cdk2 *mutant clones were very small, even when clones were induced in the *Minute *background. This is consistent with the critical role of Cyclin E/Cdk2 kinase activity for DNA replication. It is likely that the residual amount of Cdk2 protein was sufficient for a few rounds of cell proliferation to give rise the small *cdk2 *mutant clones. As shown in Fig. 1A–C, removal of Cdk2 completely blocks BrdU incorporation in the SMW (19 clones examined). These results indicate that Cyclin E/Cdk2 kinase activity is absolutely required for S-phase entry in SMW. Interestingly, staining eye discs with an antibody against cyclin E revealed that Cyclin E protein accumulated to a very high level in the *cdk2 *mutant cells (Fig. 1D–F), even though no BrdU incorporation was observed in those cells. These observations indicate that high level of Cyclin E protein does not always indicate high Cyclin E/Cdk2 kinase activity, which is essential for S phase regulation. It should be pointed out that inhibition of Cyclin E kinase activity by overexpression of Dap also led to inhibition of S phase and accumulation of Cyclin E message and protein [12,18].

### Simultaneous expression of Cyclin E and Cdk2 can overcome the G1 arrest in SMW from removal of Su(H)

Notch signaling has been shown to be required for S phase entry in the SMW. S phase cells were not observed in the SMW when Notch signaling was blocked by removal of either Notch receptor or Su(H) transcription factor (Fig. 1G–I and [9,10]). However, the level of Cyclin E was not reduced in the SMW and remained accumulated posterior to the SMW in the *Su(H) *mutant clones (Fig. 1J–L and [9,10]). These observations led to the suggestion that Cyclin E was not required for Notch mediated S phase regulation in SMW [9,10].

Since our results suggested that Cyclin E/Cdk2 activity is absolutely required for S phase in the SMW and that Cyclin E protein levels do not always correspond to Cyclin E-dependent kinase activity, we initiated experiments using the MARCM system to determine if expressing Cyclin E and its kinase partner Cdk2 either alone or together in *Su(H) *mutant clones can restore S phase in the SMW. As shown in Fig. 1M–O, while expressing either Cyclin E alone or Cdk2 alone in *Su(H) *mutant cells was not sufficient to restore S phase, expressing Cyclin E together with Cdk2 led to extensive S phase entry in Su(H) mutant clones spanning the SMW (Fig. 1M–O). These results indicated that insufficient Cyclin E/Cdk2 kinase activity is responsible for the inability of *Su(H) *mutant cells to enter S phase in the SMW, and that expression of both Cyclin E and Cdk2 are required to sufficiently increase the Cyclin E/Cdk2 kinase activity to drive *Su(H) *mutant cells to enter S phase.

### Cell cycle arrest of Su(H) mutant cells in the SMW is mediated in part by up-regulated Dacapo expression

**Figure 2:**
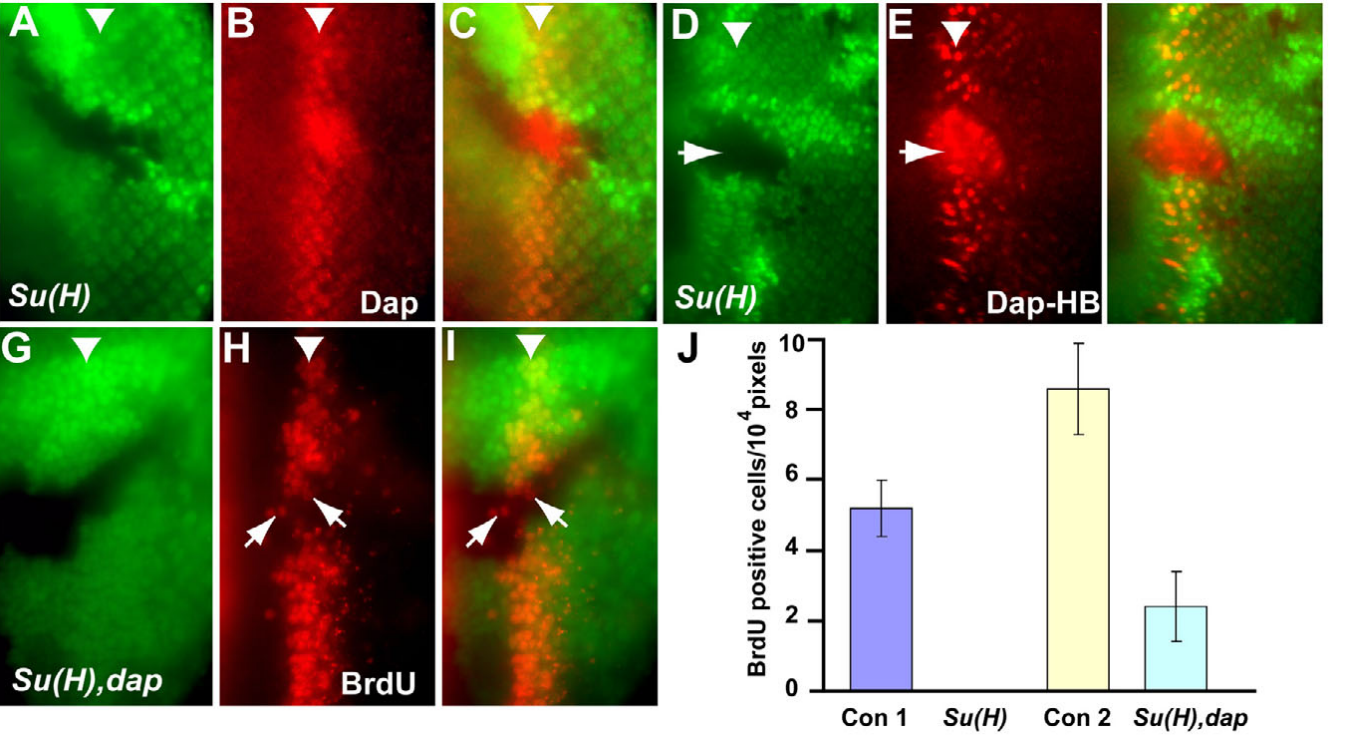
**Dap is upregulated in *Su(H) *mutant clones spanning the SMW and contributes to the block of S phase entry in the SMW**. (A-C) Dap protein (red) is up-regulated in *Su(H) *mutant cells spanning the SMW. (D-F) Dap-HB enhancer activity (red) is also upregulated in *Su(H) *mutant clones spanning SMW. (G-I) In *dap *mutant background there is a significant number of *Su(H) *mutant cells incorporating BrdU near SMW (red). Mutant clones are mark by absence of GFP and the arrows mark some BrdU cells in *Su(H)*, *dap *double mutant clone. (J) Quantification of the number of *Su(H) *mutant cells incorporating BrdU in the WT (*Su(H)*) or *dap *mutant background (*Su(H)*, *dap*). The average number of BrdU incorporating cells is normalized to the unit area (10^4 ^pixels) and Con 1 and Con 2 indicate the average number of BrdU incorporating cells in the SMW of WT or *dap *eye discs adjacent to the mutant clones.

The above results raised the question of how Cyclin E/Cdk2 kinase activity is inhibited in *Su(H) *mutant cells. Cyclin E/Cdk2 kinase activity is negatively regulated by the cyclin-dependent kinase inhibitor Dap. Dap protein has been shown to bind and inhibit the activity of Cyclin E/Cdk2 and maintain G1 arrest [11,14,19]. To determine the effect of removing *Su(H) *on Dap expression, the level of Dap protein was determined by immunostaining in eye discs containing *Su(H) *mutant clones. As shown in Fig. 2A–C, an increased level of Dap protein was observed in *Su(H) *mutant cells in the SMW. To determine if a transcriptional or posttranscriptional mechanism is involved in the observed upregulation of Dap protein in *Su(H) *mutant cells, the effect of *Su(H) *mutation on an eye disc enhancer of Dap, Dap-HB, was examined. As shown in Fig. 2D–F, increased expression of β-gal reporter from the Dap-HB enhancer was observed in *Su(H) *mutant clones in the SMW (Fig. 2D–F), indicating that the observed upregulation of Dap protein levels is mediated at the level of transcription. We therefore conclude that cell cycle arrest of *Su(H) *mutant cells in the SMW correlated with up-regulation of Dap expression, which can inhibit Cyclin E/Cdk2 activity.

To determine whether increased Dap expression actually contributes to the observed cell cycle arrest of *Su(H) *mutant cells in the SMW, we generated *Su(H) *mutant clones in *dap *mutant background and carried out a BrdU incorporation assay (Fig. 2G–I). While no BrdU incorporation was observed in *Su(H) *mutant clones spanning the SMW (Fig. 1G–I, [9]), a significant number of *Su(H)*, *dap *double mutant cells near the SMW had incorporated BrdU (Fig. 2G–J). An average of 2.4 *Su(H) *mutant cells was observed to be in S phase in an area of 10^4 ^pixels in *dap *mutant background. This compares with an average of 8.6 S phase cells that were observed in the same size area adjacent to the mutant clones (Fig. 2J). These results indicate that Dap expression contributes the G1 cell cycle arrest of *Su(H) *mutant cells. The presence of a high level of Cdk inhibitor Dap in *Su(H) *mutant clones likely provides a very high barrier for the activation of Cyclin E/Cdk2 kinase activity. This potentially explains why both Cyclin E and Cdk2 need to be overexpressed in *Su(H) *mutant cells to overcome the G1 cell cycle arrest. As not all of the *dap*, *Su(H) *mutant cells within the SMW incorporated BrdU, it is likely that additional mechanisms, such as inhibition of E2F transcription factor by RBF, also contribute to the observed cell cycle arrest of *Su(H) *mutant cells [9,10]. Indeed, RBF was shown to function redundantly with Dap in mediating the cell cycle arrest in the MF [9].

To determine if Dap may contribute to the G1 cell cycle arrest in SMW when the Notch receptor was removed, we analyzed the level of Dap in *notch *mutant clones that span the SMW. Interestingly, reduced level of Dap was observed in *notch *mutant clones ([10] and data not shown). These observations suggest that Dap accumulation is unlikely to contribute to the G1 arrest of *notch *mutant cells and that the G1 cell cycle arrest of *notch *and *Su(H) *mutant cells in the SMW probably involve distinct mechanisms.

### bHLH protein Da is required for Dap up-regulation in Su(H) mutant cells

Increased Dap expression in *Su(H) *mutant clones could be mediated directly through a repression of Dap expression by Su(H) or indirectly through the control of other transcription factor. We have shown previously that Dap-HB enhancer is regulated by bHLH proteins Ato/Da and the Ets protein Pointed (Pnt), which bind to the E-box and Ets binding sites, respectively [15]. In contrast, no Su(H) binding site was observed in the Dap-HB enhancer. Because Ato was shown to be upregulated in *Su(H) *mutant clones near the MF and because overexpression of Ato and Da in the posterior of the eye disc was sufficient to induce Dap-HB expression [15], we tested the requirement of bHLH protein Da for the upregulation of Dap in *Su(H) *mutant clones.

**Figure 3:**
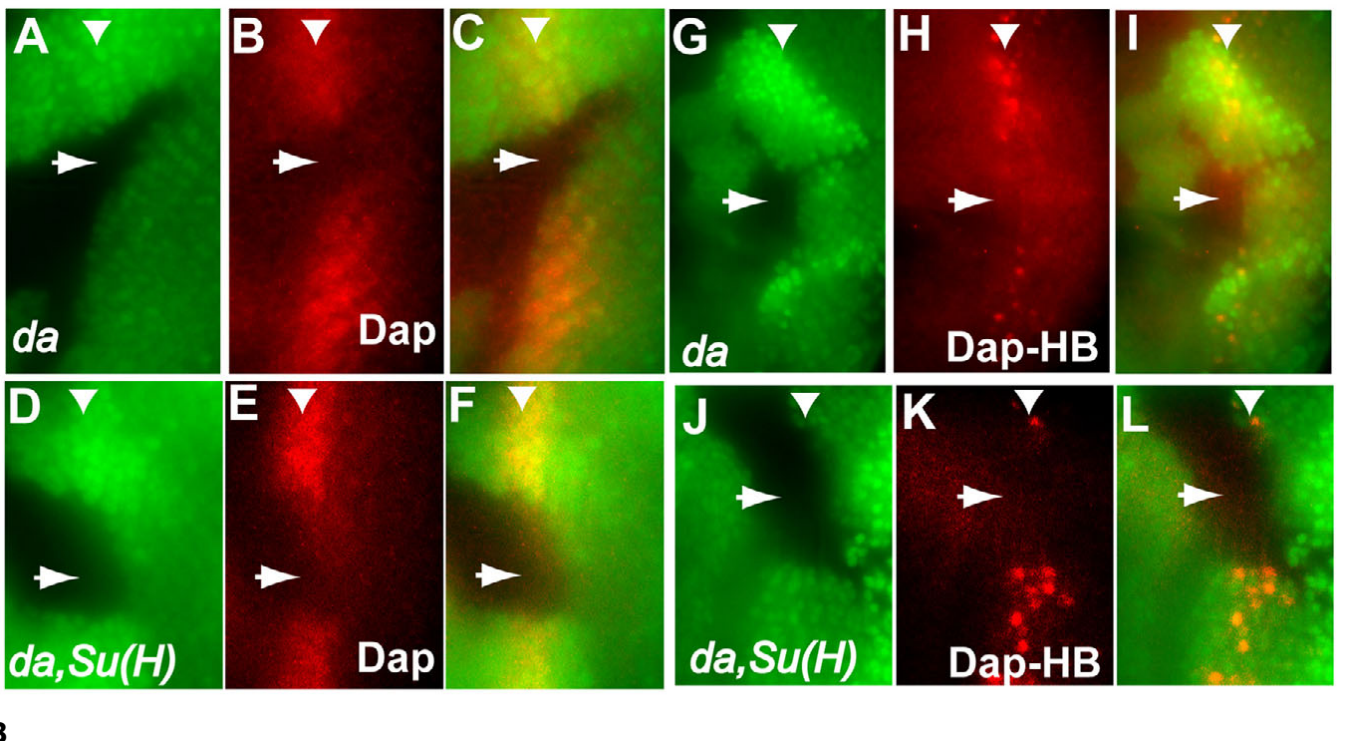
**Daughterless is required for the endogenous Dap expression and for the ectopic Dap expression in *Su(H) *mutant cells**. The level of Dap protein (red) is decreased in *da *mutant clones (A-C) as well as in *da*, *Su(H) *double mutant clones (D-F). No Dap-HB reporter activity (red in G-I) was observed in *da *mutant clones. Although increased Dap-HB reporter expression was detected in the *Su(H) *single mutant clones (Fig. 2D-F), no increased reporter activity was observed in *da*, *Su(H) *double mutant clones (red in J-L). Mutant cells are marked by the absence of GFP.

As reported earlier, Dap protein and expression was upregulated in the MF and the SMW in the developing eye [11,14,16]. This Dap expression is dependent upon the bHLH protein Da, as Dap protein as well as Dap-HB enhancer activity was significantly reduced in *da *mutant clones (Fig. 3A–C and 3G–I, [15]). To determine whether Da is also required for up-regulation of Dap expression in *Su(H) *mutant clones, we generated *da*, *Su(H) *double mutant clones. Both the level of Dap protein and the level of Dap-HB reporter were greatly reduced in the *da*, *Su(H) *double mutant cells (Fig. 3D–F,J–L), indicating that Da is required for the observed up-regulation of Dap in *Su(H) *mutant clones. We conclude from these observations that the bHLH protein Da is also required for the upregulation of Dap in *Su(H) *mutant clones.

The above results show that Cyclin E/Cdk2 kinase activity is essential for S phase in the SMW and that the G1 arrest of *Su(H) *mutant cells in the SMW is mediated by an inhibition of the Cyclin E/Cdk2 activity, in part through an upregulation of Dap expression. As Dap expression was shown to be often coordinately regulated with cell type specification by the same developmental mechanisms [15], it is likely that the role of Notch signaling in S phase regulation in the SMW is also coordinated with its role in differentiation. As Cyclin E/Cdk2 activity is critical for S phase regulation in SMW, we predict that the G1 arrest observed in *notch *mutant clones will also involve an inhibition of Cyclin E/Cdk2 activity. The decreased level of Cyclin A protein in the absence of *notch *mutants [10] could be a reflection of inhibited Cyclin E/Cdk2 activity since Cyclin A protein is destabilized by Roughex (Rux), which is in turn down-regulated by Cyclin E activity [20,21]. However, it should be pointed out that the G1 cell cycle arrest of *notch *mutant clones does not involve Dap induction and we have not tested the effect of expressing Cyclin E/Cdk2 in the *notch *mutant clones due to technical difficulties. Therefore it is formally possible that the G1 arrest observed in *notch *mutant clones does not involve an inhibition of the Cyclin E/Cdk2 activity. Further studies will be needed to determine whether Cyclin E/Cdk2 activity is inhibited in *notch *mutant clones.

### Dap is important for timely cell cycle exit from the SMW in developing eye

The observation that Dap is partially responsible for the G1 cell cycle arrest of the *Su(H) *mutant cells prompted us to carefully examine the role of Dap in cell cycle exit in the developing eye. Previous published reports did not find obvious cell cycle defects in *dap *mutant clones in the developing eye, and adult escapers of *dap *mutants did not show dramatic eye developmental defects [9,11]. These observations led to the idea that Dap did not play an important role in cell proliferation in the developing eye disc. However, our analysis described below show that Dap is required for normal cell cycle exit from the SMW.

**Figure 4:**
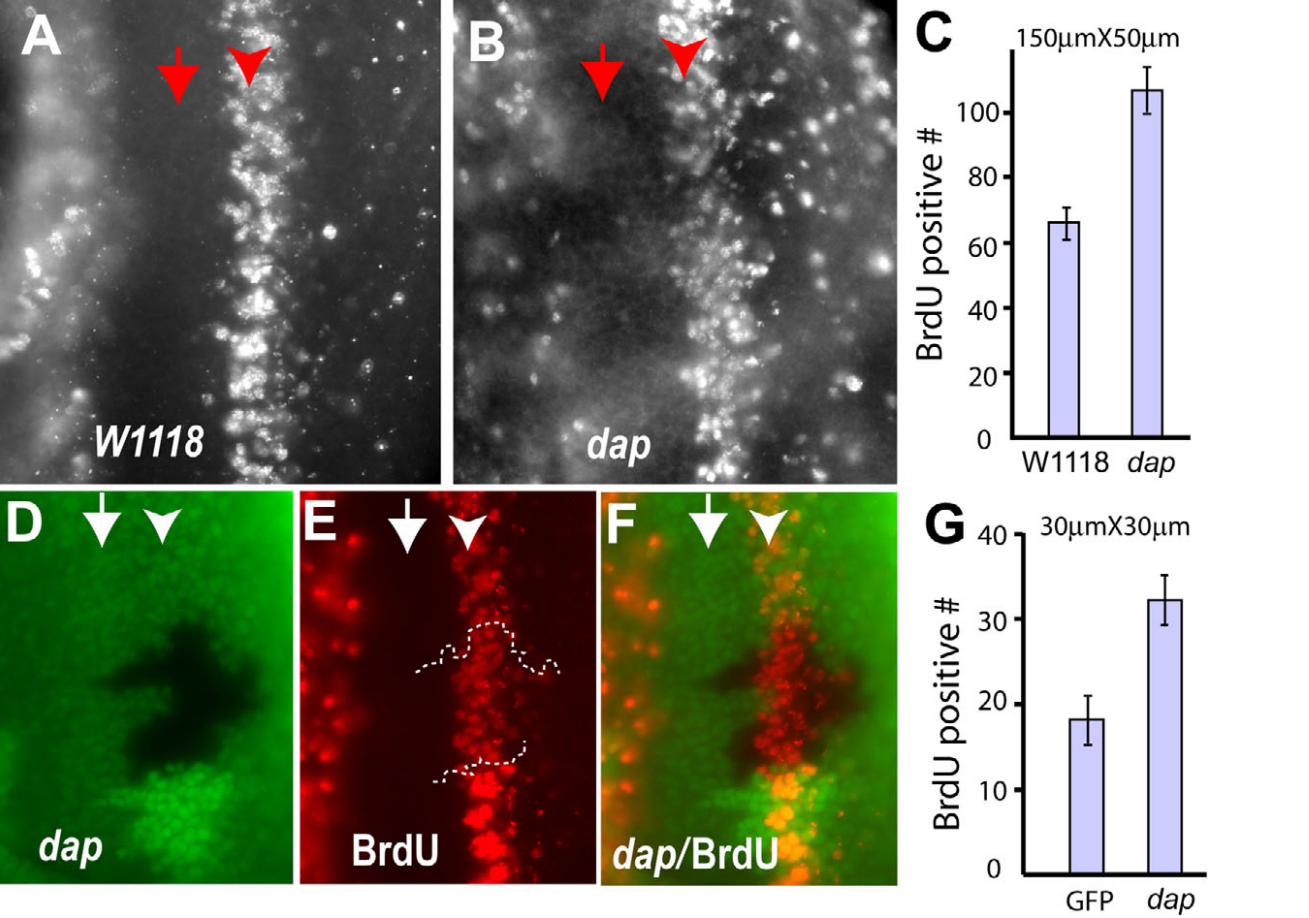
**Dap is important for normal cell cycle arrest of cells exiting the SMW**. BrdU incorporation of *w*^*1118 *^(A), *dap *mutant (B) eye discs, and eye discs with *dap *mutant clones (D-F). (C) Quantification of BrdU positive cells in *w*^*1118 *^and *dap *mutant eye discs. BrdU positive cells within an area of 150 μm × 50 μm along the SMW from 5 eye discs were counted. (G) Quantification of BrdU positive cells within a 30 μm × 30 μm area in *dap *mutant clones or adjacent WT tissues. 10 independent *dap *mutant clones and adjacent WT tissues were analyzed.

Careful examination of BrdU incorporation in *dap *mutant eye discs revealed that while no BrdU incorporation was observed within the MF in *dap *mutants, the SMW in *dap *mutant eye discs was broader and increased BrdU incorporation was observed posterior to the SMW (Fig. 4A,B). Quantification of the number of BrdU positive cells in a 50 μm × 150 μm area around the SMW in WT and *dap *mutant eye discs were 66 ± 5 and 107 ± 7, respectively (Fig. 4C, P < 1 × 10^-8^, n = 10 for WT discs and n = 5 for *dap *mutant discs). Similarly, an increased number of BrdU positive cells near SMW was observed in *dap *mutant clones as compared to adjacent WT tissues (Fig. 4D–F). The number of BrdU positive cells in a 30 μm × 30 μm area around the SMW in *dap *mutant clones and the adjacent cells were 31.8 ± 3.0 and 17.9 ± 2.7, respectively (Fig. 4G, n = 10, P < 3.4 × 10^-7^). These observations indicated that while loss of Dap did not lead to ectopic S phase entry in the MF, it did play an important role in the cell cycle exit from the SMW.

Photoreceptors R8, R2, R5, R3, and R4 are determined as cells exit the MF, and they do not undergo another round of cell proliferation, while photoreceptors R1, R6, R7, cone cells, pigment cells, and bristle cells are derived from the SMW cells. To determine whether photoreceptor cells or non-photoreceptor cells enter additional cell cycles in the absence of Dap, we carried out Elav-BrdU double-labeling of *dap *mutant eye discs. No co-localization of Elav and BrdU was observed (data not shown), indicating that the ectopic BrdU incorporation observed in the absence of Dap was from the non-photoreceptor cells. These observations are consistent with the previous reports that Dap and RBF act redundantly in controlling cell cycle arrest in the MF [9,22].

**Figure 5:**
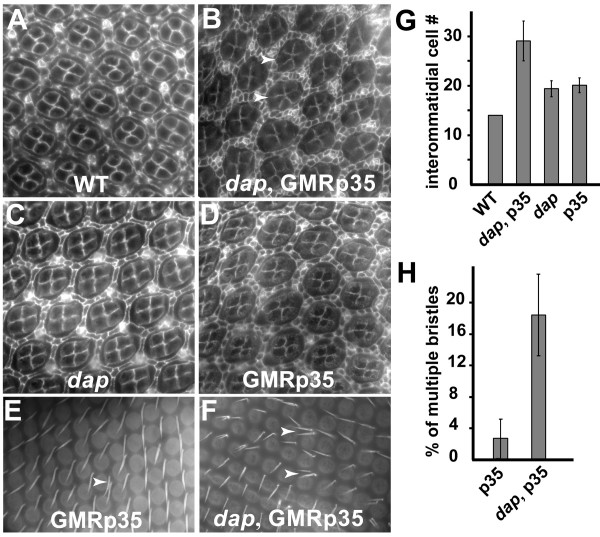
**Dap mutant eye discs have increased number of interommatidial cells and bristles**. Apical profiles of cells in the 48 h (A-D) and 55 h (E-F) APF pupal retinas stained with anti-Disc large antibodies. (A) Wild type retina. (B) *dap *mutant retina expressing the caspase inhibitor p35 under the control of GMR promoter (GMR-p35). (C) *dap *mutant retina. (D) Wild type expressing GMR-p35. (G) Quantification of the average number of interommatidial cells in WT, *dap*, GMR-p35, and *dap *mutant retinas expressing GMR-p35 is shown. (E-F) Phalloidin staining of pupae retina showing significantly more multiple bristles in *dap *mutant pupal retina expressing GMR-p35 (E) than in wild type retina expressing GMR-p35 (F). (H) Quantification of the incidence of multiple bristles in different genotype pupae discs is shown.

An additional round of cell proliferation in the SMW and the posterior is expected to generate extra cells. However, developmentally regulated apoptosis during pupae stage after the completion of cone cell, pigment cell and bristle specification generally eliminates extra cells [3]. This developmentally regulated apoptosis potentially contributes to the relatively normal adult eye structures of *dap *mutant escapers. To further characterize the consequences of extra cell proliferation in *dap *mutant eye discs, we co-expressed the apoptosis inhibitor, baculovirus p35, in the posterior of the eye disc using the GMR driver (GMR-p35) and examined pupal eye discs 40–44 hr after puparium formation (APF). As shown in Fig. 5, some of the ommatidia in *dap*, *GMR-p35 *eye discs exhibit 5 cone cells instead of the normal 4 cone cells observed in GMR-p35 flies (Fig. 5A–D). In addition, blocking apoptosis by expressing GMR-p35 in *dap *mutant background leads to significantly larger interommatidial spaces than either expressing GMR-p35 or mutations of *dap *alone (Fig. 5A–D). Quantification of cells in the interommatidial spaces revealed that there were significantly more interommatidial cells per cluster in *dap *mutant discs than in *w1118 *discs (Fig. 5G). The average number of those cells in *w1118 *and *dap *mutant pupal eyes were 14 ± 0 (n = 23) and 19.4 ± 1.6 (n = 41), respectively (P < 1.8 × 10^-23^). Moreover, inhibition of apoptosis by expressing GMR-p35 resulted in a further increase in the number of interommatidial cells. The number of interommatidial cells in *dap*, *GMR-p35 *and *GMR-p35 *pupae retina were 29 ± 4 (n = 31) and 20.1 ± 1.5 (n = 22) per cluster, respectively (P < 6.4 × 10^-13^). Furthermore, we also compared the bristle phenotype of *dap*, *GMR-p35 *pupal retina with that of *GMR-p35 *at 52–55 h APF. Compared to the *GMR-p35 *pupal retinas, the incidence of multiple bristles in *dap*, *GMR-p35 *pupae retina was about 7 times higher (Fig. 5E,F,H). These observations demonstrated that loss of Dap did indeed lead to extra cell proliferation of the non-photoreceptor cells, and that the consequences of extra cell proliferation in the absence of Dap were partially offset by apoptosis.

The observed effect of removing Dap from cells undergoing cell cycle exit from the SMW in the developing eye disc is reminiscent of the effect of loss of Dap in cells undergoing cell cycle exit after mitosis 16 in the epidermis during embryogenesis. In both cases, the high level of Dap expression in cells undergoing the last round of proliferation was required for their normal cell cycle exit, and removing Dap led to additional cell proliferation [11,14]. In addition, RBF was required to maintain cell cycle arrest of both the postmitotic epidermal cells and the non-photoreceptor cells in the posterior of the developing eye disc [9,23,24]. The observed requirements for both Dap and RBF for the cell cycle arrest of these cell types is in stark contrast to the requirement of Dap and RBF for the cell cycle arrest of the differentiating photoreceptor cells, where either Dap or RBF is sufficient for their cell cycle arrest [9]. The nature of the differences in their cell cycle control is currently not known and will require further investigation.

## Conclusion

Our results demonstrated an essential role for the Cyclin E/Cdk2 kinase activity in S phase regulation in SMW and an important role for Dap in the normal G1 arrest of cells exiting the SMW. In addition, we showed that Dap expression, which requires the bHLH protein Da, is induced in *Su(H) *mutant cells near SMW and contributes to their cell cycle arrest.

## Methods

### Fly strains and Antibodies

The following fly strains were used in this study: *Su(H)*^*Δ47 *^[5], Dap-HB-lacZ [16], UAS-Ato and UAS-Da [25], *dap*^*4*^, *dap*^*4454 *^[11,14], *da*^*10 *^[26]. The following antibodies were used. Mouse anti-cycE (gift of H. Richardson), mouse monoclonal anti-Dap (gift of I. Hariharan), rabbit anti-Ato (gift of Y.N. Jan). Mouse monoclonal anti-β-galactosidase (mAB40-1a), mouse anti-Disc large, and rat anti-Elav were obtained from the Developmental Studies Hybridoma bank at the University of Iowa. The genotypes used in this study are listed below:

Df(2L)da^10 ^FRT40A/UbiGFP FRT40A

*Df(2L)da^10 ^FRT40A/UbiGFP FRT40A; Dap-HB-lacZ/*+

Su(H)^Δ47 ^FRT40A/UbiGFP FRT40A

*Su(H)^Δ47 ^FRT40A/UbiGFP FRT40A; Dap-HB-lacZ/*+

dap^4 ^Su(H)^Δ47 ^FRT40A/dap^4454 ^UbiGFP FRT40A

Df(2L)da^10 ^Su(H)^Δ47 ^FRT40A/UbiGFP FRT40A

Df(2L)da^10 ^Su(H)^Δ47 ^FRT40A/UbiGFP FRT40A; Dap-HB-lacZ/+

dap^4^/dap^4454^

FRT42B dap^4^/Ubi GFP FRT42B

UAS-Ato UAS-Da/UAS-GFP Act>CD2>Gal4

FRT42B dap^4^/dap^4454^; UAS-Ato UAS-Da/UAS-GFP Act>CD2>Gal4

### BrdU incorporation, Phalloidin staining, and Immunohistochemistry

Eye discs were dissected, incubated with BrdU (75 μg/ml final) at RT for 60 min, washed with PBS, and fixed with 4% paraformaldehyde in PBS followed by post fix with 4% paraformaldehyde in PBS+0.6% Tweeen-20. The discs were washed with DNase I buffer followed by incubation with DNase I (100 U/500 μl) for 1 hour. Mouse anti BrdU antibody (Becton Dickinson) was used at 1:50 dilution. Immunohistochemistry and Phalloidin staining were performed essentially as described [24].

## Authors' contributions

MS carried out the genetics, immunofluorescence studies, quantative analysis, and drafted the manuscript. WD participated in the design and coordination of this study and in the writing of this manuscript. All authors read and approved the final manuscript.
